# Integrating explainable deep learning with multi-omics for screening progressive diagnostic biomarkers of hepatocellular carcinoma covering the “inflammation-cancer” transformation

**DOI:** 10.1016/j.jpha.2025.101253

**Published:** 2025-03-01

**Authors:** Saiyu Li, Yiwen Zhang, Lifang Guan, Yijing Dong, Mingzhe Zhang, Qian Zhang, Huarong Xu, Wei Xiao, Zhenzhong Wang, Yan Cui, Qing Li

**Affiliations:** a School of Pharmacy, Shenyang Pharmaceutical University, Shenyang, 110016, China; b State Key Laboratory of New-tech for Chinese Medicine Pharmaceutical Process, Jiangsu Kanion Pharmaceutical Co., Ltd., Lianyungang, 222001, China

## Abstract

• The lipids fingerprint profile from the inflammation to cancer in HCC was provided.
• Applying ML and explainable DL to analyze multi-omics data showed great potential.
• 12 lipid biomarkers associated with “inflammatory-cancer” transition were identified.
• A robust diagnostic model was developed for predicting HCC progression.

## Graphical abstract

**Figure:**
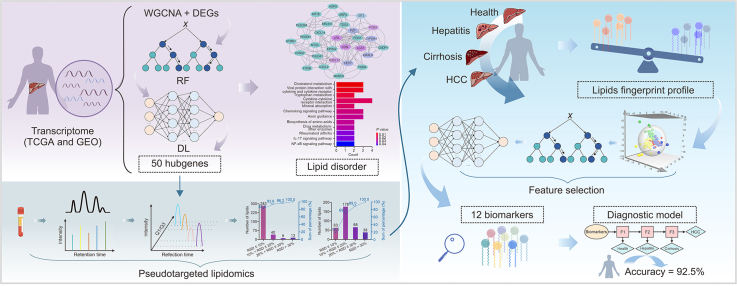

Chronic uncontrolled inflammation is a major risk factor driving the occurrence of hepatocellular carcinoma (HCC), with over half of global cases attributed to hepatitis B virus (HBV) infection. Persistent inflammation frequently progresses to cirrhosis and, ultimately, malignancy [1]. Monitoring the key risk factors involved in the inflammatory-to-cancerous transformation in HCC is crucial for enabling timely intervention and improving patient survival rates. To address this challenge, we analyzed plasma samples collected from healthy volunteers and patients at various stages of HCC progression, including hepatitis, cirrhosis, and HCC (Approval No.: 2021-IRBQYYS-021) (Tables S1–S5). We employed a progressive biomarker screening methodology integrating classical machine learning (ML) and interpretable deep learning (DL) techniques to analyze and interpret multi-omics data. This multi-omics analysis provided insights into the pathological mechanisms underlying HCC [2], while the advanced capabilities of ML and DL approaches enhanced the accuracy of feature extraction [3,4]. Ultimately, we identified 12 plasma metabolic biomarkers and developed a high-performance diagnostic model to monitor HCC progression. This model is expected to become a potential tool in clinical practice.

The transcriptome data from The Cancer Genome Atlas (TCGA) database were initially analyzed to elucidate the pathophysiological mechanisms underlying HCC. The workflow is depicted in Supplementary data and Fig. 1A. First, we performed weighted gene co-expression network analysis (WGCNA) and differential expression gene (DEG) analysis to identify 250 critical genes (Figs. S1A–F). Subsequently, random forest (RF) and DL models (Tables S6 and S7) were constructed to further pinpoint 50 hub genes with a mean absolute Shapley Additive exPlanation (SHAP) value > 0.15 [5]. The receiver operating characteristic (ROC) curves of these models is shown in Fig. S1G. Kyoto Encyclopedia of Genes and Genomes (KEGG) pathway enrichment analysis revealed that these 50 hub genes were particularly abundant in cholesterol metabolism (Fig. 1B). Next, we applied the maximum clique centrality (MCC) algorithm to analyze the protein-protein interaction (PPI) network, identifying hub genes with the top 3 MCC scores, including *LPA*, *LCAT*, and *CD5L*, which are associated with lipid metabolism (MCC scores > 20) (Fig. S1H). The expression levels of these genes were significantly downregulated in HCC tumor tissues according to both the TCGA and Gene Expression Omnibus (GEO) datasets (GSE174570) (Figs. 1C, 1D, and S1I–S1L). Collectively, these findings suggest that disruptions in lipids metabolism accompany the development of HCC.

**Fig. 1:**
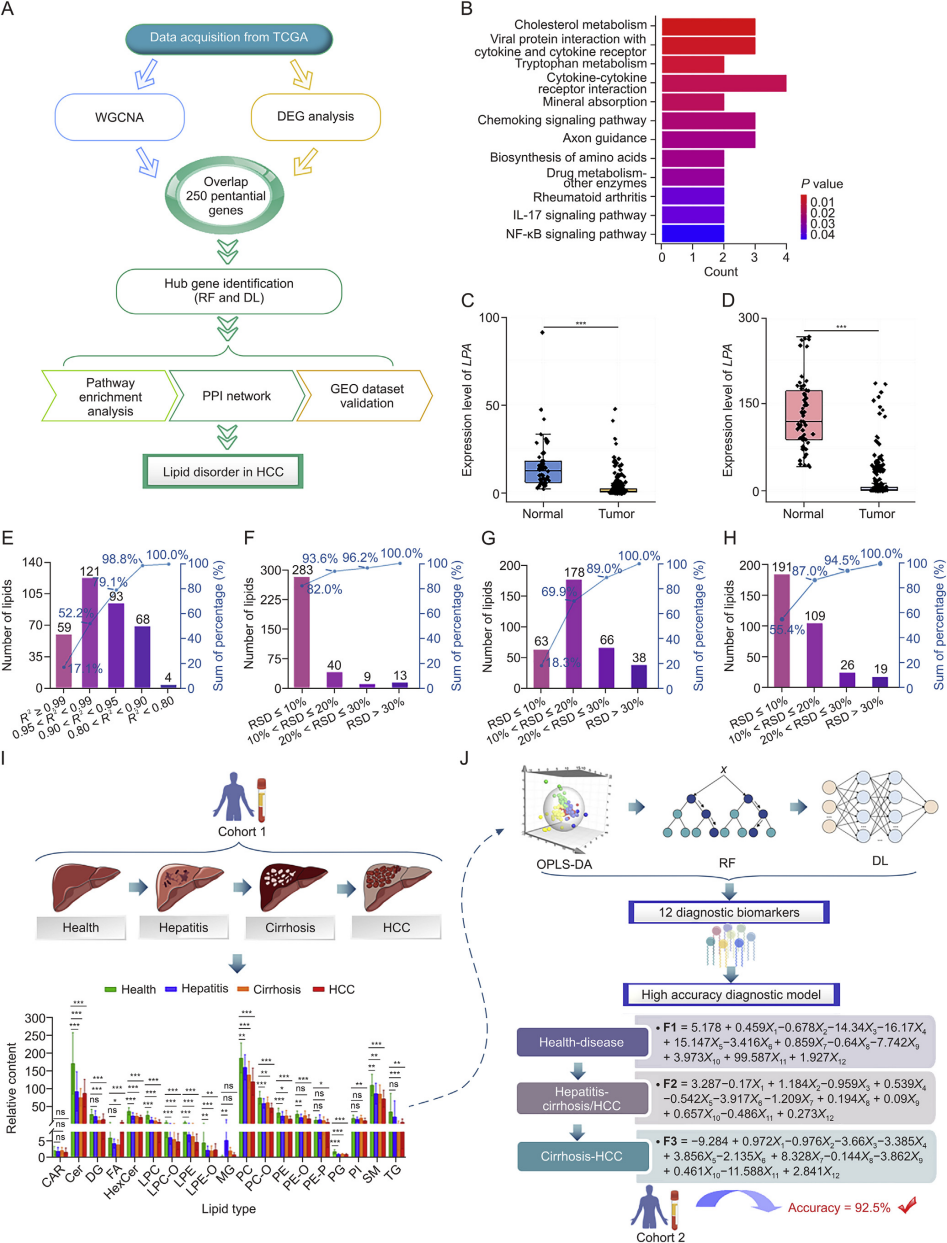
Overview of the workflow for screening progressive biomarkers in the “inflammation-cancer” transformation pattern of hepatocellular carcinoma (HCC). (A) The workflow for screening hub genes and exploring potential pathological mechanisms of HCC formation. (B) Kyoto Encyclopedia of Genes and Genomes (KEGG) pathway enrichment analysis. (C, D) Expression levels of the *LPA* gene in The Cancer Genome Atlas (TCGA) database (C) and GSE174570 from the Gene Expression Omnibus (GEO) database (D). (E–H) Linearity (E), intra-day repeatability (F), inter-day repeatability (G), and stability (H) in positive ion mode of the pseudotargeted lipidomics method. (I) The relative total content of individual lipid subclasses revealed that lipids underwent continuous metabolic alterations during the “inflammation-cancer” transformation of HCC. (J) Schematic diagram showing the integration of orthogonal partial least squares-discriminant analysis (OPLS-DA), random forest (RF), and deep learning (DL) models to identify 12 progressive diagnostic biomarkers and establish a diagnostic model for HCC progression. ^∗^*P* < 0.05, ^∗∗^*P* < 0.01, and ^∗∗∗^*P* < 0.001. ns: no significance. WGCNA: weighted gene co-expression network analysis; DEG: differential expression gene; PPI: protein-protein interaction; IL: interleukin; NF-κB: nuclear factor-kappaB; RSD: relative standard deviation; CAR: carnitine; Cer: ceramide; DG: diacylglycerol; FA: fatty acid; HexCer: hexosylceramide; LPC: lysophosphatidylcholine; LPC-O: alkyl-LPC; LPE: lysophosphatidylethanolamine; LPE-O: alkyl-LPE; MG: monoacylglycerol; PC: phosphatidylcholine; PC-O: alkyl-PC; PE: phosphatidylethanolamine; PE-O: alkyl-PE; PE-P: alkenyl-PE; PG: phosphatidylglycerol; PI: phosphatidylinositol; SM: sphingomyelin; TG: triacylglycerol.

Based on transcriptome results, we developed a pseudotargeted lipidomics protocol to fingerprint lipid metabolites during the “inflammation-cancer” transformation (Supplementary data and Fig. S2A). Initially, we established a non-targeted lipid analysis method using high performance liquid chromatography-tandem quadrupole time-of-flight mass spectrometry (HPLC-QTOF/MS) (Table S8 and Figs. S2B and C). This process generated two multiple reaction monitoring (MRM) ion candidate lists in positive (610 ions) and negative (223 ions) ion modes. These candidate ion pairs were verified and optimized using HPLC-triple quadrupole MS (HPLC-TQMS), resulting in the retention of 345 and 110 ion pairs in positive and negative ion modes, respectively (Table S9 and Figs. S2D and E). Subsequently, plasma quality control (QC) was performed to validate the analytical method. The results exhibited that over 98.8% of the lipids demonstrated good linearities (*R*^2^ ≥ 0.8; Figs. 1E and S2F). The intra-day and inter-day repeatability analysis revealed that 96.2% and 89.0% of analytes in positive ion mode, and 100.0% and 96.4% in negative ion mode had relative standard deviations (RSDs) < 30% (Figs. 1F, 1G, S2G, and S2H). Stability evaluation indicated that 87.0% and 95.5% of the analysts had RSD < 20% in positive and negative ion modes, respectively (Figs. 1H and S2I). Based on these robust results, the developed pseudotargeted lipidomics method is suitable for large-scale lipid analysis of plasma.

The well-established pseudotargeted lipidomics method was applied to characterize lipid fingerprint profiles throughout the progression from “health-hepatitis-cirrhosis-HCC” (Fig. 1I). The analysis of lipid subclasses revealed dynamic metabolic changes associated with HCC progression, highlighting the interaction between metabolic shifts and pathological evolution during the “inflammation-cancer” transformation.

We constructed a strategy that integrated classical ML with interpretable DL to identify lipid biomarkers serving as precise indicators of the “inflammation-cancer” transformation (Fig. 1J). Orthogonal partial least squares discriminant analysis (OPLS-DA) demonstrated significant perturbations in lipid metabolism throughout this transformation (Figs. S3A and B), and 51 features with variable importance in projection (VIP) > 1 were selected for further analysis. Subsequently, RF and explainable DL models were trained using the selected features from cohort 1 to rigorously extract biomarkers with discriminatory capabilities (Tables S6 and S7). The performance of these models was verified using the ROC curve on the testing set (Fig. S3C). Additionally, the accuracy, precision, recall, and F1 score of the established DL model were achieved at 0.8333, 0.9000, 0.8333, and 0.8125, respectively. The robust efficacy of the DL model in diagnosing various stages of HCC progression was further validated by a 10-fold cross-validation (Fig. S3D). Ultimately, 12 lipid biomarkers were identified through sensitivity analysis (Figs. S3E–G and S4). Additional algorithms, including RF and partial least squares discriminant analysis (PLS-DA), confirmed the exceptional proficiency of these 12 biomarkers in distinguishing different stages of HCC progression (Fig. S5 and Tables S10 and S11).

To facilitate clinical application, a diagnostic model with an accuracy of 88.9% was successfully constructed to determine the progression of HCC based on the 12 biomarkers (Tables S12 and S13). This diagnostic model demonstrated a robust prediction accuracy of 92.5% in an independent validation cohort 2 (Table S14). The ROC curves and other performance metrics of the diagnostic model are presented in Fig. S6 and Table S13, respectively.

This study revealed that the dynamic modifications in lipid profiles are associated with the progression of HCC, as evidenced by both transcriptomics and lipidomics. These findings offer a novel perspective on intervening in the formation of HCC by regulating lipid metabolism or the corresponding enzymes. Our findings underscore the distinctive benefits of integrating ML and explainable DL with multi-omics analysis to identify crucial biomarkers and address complex problems. Notably, the diagnostic model we developed enhances the prediction of HCC progression risk and holds considerable promise in meeting a significant unmet medical need.

## CRediT authorship contribution statement

**Saiyu Li:** Writing – review & editing, Methodology, Conceptualization. **Yiwen Zhang:** Writing – review & editing, Methodology, Conceptualization. **Lifang Guan:** Visualization, Software, Data curation. **Yijing Dong:** Visualization, Software, Data curation. **Mingzhe Zhang:** Validation, Investigation. **Qian Zhang:** Validation, Investigation. **Huarong Xu:** Validation, Investigation. **Wei Xiao:** Supervision, Investigation. **Zhenzhong Wang:** Supervision, Investigation. **Yan Cui:** Writing – review & editing, Supervision, Funding acquisition. **Qing Li:** Writing – review & editing, Supervision, Funding acquisition.

## Declaration of competing interest

The authors declare that there are no financial interests or personal relationships that may be considered as potential competing interests: authors Wei Xiao and Zhenzhong Wang served as Chairman and General Manager of Jiangsu Kanion Pharmaceutical Co., Ltd., respectively, during the research. Moreover, Jiangsu Kanion Pharmaceutical Co., Ltd. has no relevant relationships or competing interests related to this research.
