# Supplementary material for: Integrating explainable deep learning with multi-omics for screening progressive diagnostic biomarkers of hepatocellular carcinoma covering the “inflammation-cancer” transformation
Source: J Pharm Anal. 2025 Mar 1;15(9):101253. doi: 10.1016/j.jpha.2025.101253 (PMC12410188; doi:10.1016/j.jpha.2025.101253)
Supplement: Multimedia component 1 [file mmc1.docx]

Supplementary Material

Integrating explainable deep learning with multi-omics for screening progressive diagnostic biomarkers of hepatocellular carcinoma covering the “inflammation-cancer” transformation

Table of Contents:

**1. Experimental section**

**2. Fig. S1.** Identifying hub genes and elucidating the potential pathological mechanisms underlying hepatocellular carcinoma (HCC) development.

**3. Fig. S2.** Development and validation of pseudotargeted lipidomics method.

**4. Fig. S3.** Screen for progressive diagnostic biomarkers to indicate the progression of hepato-cellular carcinoma (HCC).

**5. Fig. S4.** The expression levels of 12 lipid biomarkers across the health, hepatitis, cirrhosis, and hepatocellular carcinoma (HCC) groups.

**6. Fig. S5.** Receiver operating characteristic (ROC) curves of prediction results based on 12 biomarkers using random forest (RF) and partial least squares discriminant analysis (PLS-DA) models.

**7. Fig. S6.** Receiver operating characteristic (ROC) curves for the various models.

**8. Table S1.** Characteristics of the subjects.

**9. Table S2.** Correlation coefficient of age sex, and the patient disease type.

**10. Table S3.** Two-sample T test of sex and age of patients

**11. Table S4.** Two-sample T test of age and the disease type of patients

**12. Table S5.** Two-sample T test of sex and the disease type of patients

**13. Table S6.** Hyperparameters of random forest (RF) models.

**14. Table S7.** Optimization hyperparameters of deep learning (DL) models.

**15. Table S8.** HPLC-QTOF/MS parameters.

**16. Table S9.** HPLC-TQMS parameters.

**17. Table S10** Confusion matrix of prediction results based on 12 biomarkers using random forest (RF).

**18. Table S11** Confusion matrix of prediction results based on 12 biomarkers using partial least squares discriminant analysis (PLS-DA).

**19. Table S12** Diagnosis models of “health-hepatitis-cirrhosis-HCC” based on 12 lipid biomarkers.

**20. Table S13** Performance metrics of the diagnostic model in different cohorts.

**21. Table S14** The confusion matrix of prediction results in cohort 2.

**1. Experimental section**

*1.1. Materials and Chemicals*

Liquid chromatography-mass spectrometry (LC-MS)-grade methanol (MeOH), acetonitrile (ACN), and isopropanol (IPA) were obtained from Fisher Scientific (Fair Lawn, NJ, USA). High-performance LC (HPLC)-grade methyl tert-butyl ether (MTBE) was obtained from the Shandong Yuwang Industrial Co. Ltd. (Yucheng, China). Distilled water was obtained from Wahaha Co., Ltd. (Hangzhou, China). LC-MS-grade ammonium formate (AmFm) was obtained from Sigma-Aldrich (Saint-Louis, MO, USA). Internal standards (ISs) of sphingomyelin (SM) d18:1/16:0-d31 and phosphatidylglycerol (PG) 16:0-d31/18:1 were purchased from Avanti Polar Lipids Inc. (Alabaster, AL, USA).

*1.2. Plasma sample*

Clinical plasma samples from two cohorts at different stages of hepatocellular carcinoma (HCC) progression were collected for analysis. The cohort 1 consisted of healthy volunteers (*n* = 30), hepatitis patients (*n* = 30), cirrhosis patients (*n* = 30), and HCC patients (*n* = 30). An additional validation cohort 2 included 20 healthy volunteers and 20 HCC patients. The clinical information of the participants is presented in Table S1. The correlation coefficients confirmed that there was no correlation among sex, age and the disease type (Table S2). The two-sample T test analysis confirmed that there were no significant differences between sex and age, age and the disease type of patients, and sex and the disease type of patients (Tables S3–S5). The entire experiment was approved by the Research Ethics Committee of Shanghai Seventh People’s Hospital (Ethics ID number: 2021-IRBQYYS-021), and all participants provided written informed consent.

*1.3. Model of random forest and deep learning*

An innovative approach has been developed that progressively refines critical features by integrating random forest (RF) with deep learning (DL) algorithms for processing transcriptome and lipidomic datasets. Established RF models, which comprise multiple classifier trees utilizing the Gini impurity split criterion, and subsequently select a reduced set of features based on their permutation-based importance in both transcriptome and lipidomic datasets. Following this, the DL model was constructed to further refine and select core features. Briefly, the framework of DL is a fully connected neural network consisting of an input layer, one or more hidden layers, and an output layer. The independent variable was the expression of features, serving as the model’s input layer, with the number of features determining the number of neurons. The dependent variable was the disease label, which represents the last layer of the model (output layer) and contains a single neuron. Given that the dependent variable was non-numerical, we encoded the disease label with specific numerical values. The hidden layers consist of multiple neurons, which connecting the input and output layer. In the neural network, each neuron in every layer is connected to every neuron in the subsequent layer. This structure allows each neuron to receive inputs from all neurons in the previous layer, and these inputs are then weighted, summed, and then output through the activation function. In this study, Sigmoid and ReLU activation functions were used to introduce non-linear components into the models. The equations for these functions were defined as follows:

$$\text{Sigmoid}\text{:}\text{ }\text{f}\left( \text{x} \right)\text{=}\frac{1}{1+e^{-x}}$$

$$\text{Relu}\text{:}\text{ }\text{f}\left( \text{x} \right)\text{=}\left\{ \begin{aligned} -\text{x, if x≥0} \\ \text{0, if x<0 } \end{aligned} \right.$$

In these equations, x is the input signal to the neuron and *f* is the activation function.

The samples were randomly split into a training set (80%) and a testing set (20%) both in the transcriptome and lipidomics datasets. The training set was used to construct the DL classification model and the testing set was employed to assess the robust performance of the built models. Based on four recommended evaluation metrics, including accuracy, precision, recall, and F1 score, the optimal model was selected by fine-tuning hyperparameters including the hidden layers, hidden size, and activation functions. Once the DL model was finalized, its generalization capability was evaluated through tenfold cross-validation. In addition, the receiver operating characteristic (ROC) curve was applied to verify the accuracy of the established RF and DL models on the testing set. The RF and DL models were performed using the scikit-learn package (v. 1.2.0) and PyTorch package (v. 2.0.1) in Python (v. 3.10.5), respectively.

*1.4. Sensitivity analysis*

In this study, the main concept of the SHapley Additive exPlanation (SHAP) is based on cooperative game theory, which emphasizes the influence of participants on the total revenue. The combination of SHAP and DL explains the complex phenomenon of the prediction ability of the black-box model, thus revealing the relationship between the input variables and output in the model. The model produces a predictive value for each sample. The SHAP value reflects the relative contribution of each input variable to the model output, facilitating the robustness of the prediction performance. Herein, based on the activation difference of neurons in well-established DL models, backpropagation was performed on the input features, and the contribution of the input was inferred by assigning scores. The SHAP values were calculated using the SHAP package (v. 0.41.0) in Python (Version 3.10.5), and the SHAP value obeyed the following equation:

*γi=γ_base_* + *f (x_i1_) + f (x_i2_) +…+ f (x_ij_)*

In this equation, *γ_base_* is the baseline of the entire model. *f (x_ij_)* represents the contribution of the *j^th^* feature in the *i^th^* sample. *γi* refers to the predicted value of the *i^th^* sample.

*1.5. Transcriptome data analysis process*

Firstly, a quantitative profile of mRNA expression of HCC containing 50 cases of normal and 374 cases of tumors was acquired from The Cancer Genome Atlas (TCGA) database. Additionally, an mRNA expression quantification dataset (GSE174570) of HCC was obtained from the gene expression omnibus (GEO) database, which comprised 57 tumor samples and 57 normal samples. Then, the weighted gene co-expression network (WGCNA) R package (v. 1.72.5) was used to construct a gene expression matrix with 15,654 gene expression data extracted from the TCGA database. This process involved setting an appropriate soft threshold (β-value=7) to align the network with a scale-free distribution network (R^2^=0.9). Module eigengenes (MEs) were then computed to analyze gene modules, with the primary principal components of each module representing the aggregate expression profiles of all genes within that module. The adjacency matrix was converted into a topological overlap matrix (TOM), employing a TOM-based dissimilarity measure with a minimum module size of 30. Clustering modules were generated using the dynamic tree-cutting technique, with a cut height parameter set at 0.25. The association between co-expression modules and clinical traits of HCC progression was then determined. Subsequently, the “Limma” R package (v. 3.60.4) was carried out to identify differentially expressed genes (DEGs) from the TCGA genes database with |log_2_ fold change (FC) | > 1. The candidate hub genes were obtained through Veen analysis by intersecting genes from two principal modules with the identified DEGs. Further refinement was performed by implementing an RF model, prioritizing genes with a feature importance score >0.001. This was followed by the establishment of a DL model to definitively identify the hub genes. Detailed hyperparameters for the RF and DL models are provided in Table S6 and Table S7, respectively. Kyoto Encyclopedia of Genes and Genomes (KEGG) pathway enrichment analysis was conducted on the screened hub genes using DAVID (https://david.ncifcrf.gov/tools.jsp) to decipher potential mechanisms of HCC pathogenesis. Additionally, a protein-protein interaction (PPI) network was constructed using the String database (https://cn.string-db.org/), providing insights into the interactions among the hub genes. The expression of genes with top 3-ranking maximum clique centrality (MCC) values in the PPI network was compared between normal and tumor tissues using *t* test on both the TCGA and the GSE174570 databases.

*1.6. Pseudotargeted lipidomics analysis protocol*

*1.6.1. Sample preparation*

A total of 200 μL plasma was spiked with 20 μL of ISs solution (5 μg/mL of SM d18:1/16:0-d31 and 50 μg/mL PG 16:0-d31/18:1). After vortexing for 30 s, 1.3 mL of MTBE:MeOH mixed solution (5:1.5, *V/V*) was added to the mixture and vortexed for 3 min. Subsequently, the mixture was centrifuged (12,000 rpm for 5 min at 4℃) followed by a step of adding 290 μL distilled water. The supernatant of the organic phase was collected and dried under nitrogen flow. The dry extract samples were reconstituted with 200 μL of MeOH for the subsequent analysis. An equal volume of plasma was taken from each plasma sample to prepare quality control (QC) samples, which were processed using the same procedure as the plasma samples.

*1.6.2. Non-targeted lipidomics analysis using HPLC-QTOF/MS*

The LC-MS system consisting of an Agilent 1260 Infinity HPLC system (Agilent, Santa Clara, CA, USA) and a TripleTOF™ 5600^+^ MS (AB SCIEX, Foster, USA) with an electrospray ion source, was carried out for non-targeted lipidomics analysis. Chromatographic separation was performed on a Waters ACQUITY BEH C_18_ (50 mm × 2.1 mm, 1.7 μm) column at 55°C with a flow rate of 0.3 mL/min. The mobile phases consisted of (A) 10 mM AmFm in CAN:H_2_O (60:40, *V*/*V*) and (B) 10 mM AmFm in IPA:ACN (90:10, *V*/*V*). The schedule of gradient eluting was from 40% B to 44% B (0–2.0 min), 44% B to 50% B (2.0–2.1 min), 50% B to 62% B (2.1–12 min), 62% B to 99% B (12–22 min), 99% B to 40% B (22–22.1 min), 40% B (22.1–30 min). The detailed mass spectrometric parameters were presented in Table S8.

*1.6.3. Pseudotargeted lipidomics analysis based HPLC-TQMS*

The pseudotargeted lipidomics analysis experiment was conducted on an XR LC-20A Prominence HPLC system (Shimadzu, Japan) in tandem with a triple quadrupole MS (TQMS) 4000 system (AB Sciex, USA). The LC analysis conditions were consistent with the description in the section on HPLC-QTOF/MS analysis. The key operating MS parameters were shown in Table S9.

*1.6.4. The workflow of* *MRM ion pair acquisition and* *optimization*

Firstly, a QC sample with three times the volume was prepared and analyzed under the described HPLC-QTOF/MS analysis conditions at various collision energy (CE) voltages (± 15 V, ± 30 V, ± 45 V). Subsequently, the software MS-DIAL (v. 4.90) was employed to extract, align, and identify peaks from the raw MS spectra data. After performing an ion fusion step to reduce redundant ions by removing features with the characteristics of low abundance peaks, isotope peaks, and dimer ions, a peak matrix including retention time (RT), accurate masses, and MS/MS fragments was established. Then, the peak matrix was imported into multiple reaction monitoring (MRM)-Ion Pair Finder ([https://github.com/zhengfj1994/MRM_Ion_Pair _Finder/tree/master/R](https://github.com/zhengfj1994/MRM_Ion_Pair%20_Finder/tree/master/R)) to define MRM transitions. The response threshold of product ions was set to 750 Da, and the *m/z* difference between precursor and produce ion was required to be greater than 14 Da. Following the rules, an MRM transitions list containing information including precursor ion, produce ion, and CE was generated. Then, HPLC-TQMS was applied to monitor the MRM ion pairs derived from non-targeted lipidomics analysis. The product ions that appeared consistently and stably were selected by optimizing the CE to achieve the optimal detection sensitivity, and unresponsive ion pairs were deleted. After removing ion pairs with peak intensity relative standard deviation (RSD) greater than 30%, MRM ion pairs lists for sample analysis was eventually generated.

*1.6.5. Method validation and Sample analysis*

The QC samples were utilized to monitor the robust detection capabilities of the established pseudotargeted lipidomics method. The examination items were mainly focused on linearity, repeatability, and stability. For the linearity test, a series of gradient QC samples of 25 μL, 50 μL, 100 μL, 200 μL, 400 μL, and 800 μL were prepared, and the regression coefficients for each analyte were examined. To evaluate repeatability, six QC samples were prepared in parallel and injected repeatedly within a day and repeated on three consecutive days. Additionally, the intensity variations of analytes in a QC sample at six times points of 0 h, 2 h, 4 h, 8 h, 16 h, and 24 h were analyzed to confirm the stability of pseudotargeted lipidomics method.

The established pseudotargeted lipidomics method was applied to analyze clinical plasma samples from different stages of HCC. Each plasma sample was tested one time. To monitor the stability of the instrument during the run analytical batch, the QC sample was inserted into every ten test samples. The instrument precision was assessed by injecting the QC sample six times continuously before testing the actual sample in each analysis batch.

*1.6.6. Data preprocessing*

The Analyst (v. 1.6.2) software was applied to acquire the intensity information of metabolites. The relative concentration of the analytes was calculated using the equation derived from the calibration curve. The data matrix was filtered according to the “80% rule” and the “0” values were filled with the minimum value present in the entire matrix.

*1.7. Determining diagnostic biomarkers and establishing diagnostic models*

The orthogonal partial least squares discriminant analysis (OPLS-DA) was initially performed on the plasma lipids matrix to differentiate between healthy individuals and various stages of hepatitis, cirrhosis, and HCC, and variable importance in projection (VIP) ≥1 was considered a reference index to screen features with a greater contribution to grouping for subsequent analysis. Based on these selected features, an RF model with 200 classifier trees was then constructed to further refine the feature set, and the features with importance values ≥ 0.001 were retained. Subsequently, a DL model was developed through hyperparameters optimization and tenfold cross-validation to assess the final set of features. Finally, according to the sensitivity analysis, 12 key biomarkers with SHAP value > 0.15 for identifying and diagnosing HCC progression were screened. Table S6 and Table S7 provided detailed optimization hyperparameters for the RF and DL models, respectively. To validate the accuracy of the selected key features in predicting various stages of HCC progression, the additional machine learning (ML) algorithms including partial least squares discriminant analysis (PLS-DA) and RF were utilized to evaluate the predictable performance of the determined progressive biomarkers. To facilitate clinical transformation, a diagnostic model for HCC progression was eventually developed based on 12 screened features using binary logistic regression analysis.

*1.8. Statistical Analysis*

Student’s *t* test and binary logistic regression analysis were performed using SPSS 23 (SPSS Inc., USA). OPLS-DA and PLS-DA analysis were carried out using SIMCA14.0 (UmetricsAB, Umea, Sweden). Origin software was used for graphical visualization. The volcano maps of DEGs were plotted using the “Pheatmap” R package (v. 1.0.12).

**Supporting Figures:**

**Fig. S1.** Identifying hub genes and elucidating the potential pathological mechanisms underlying hepatocellular carcinoma (HCC) development. (A) Gene clustering dendrogram based on topological overlap, along with their corresponding module colors. (B) Heatmap of module-trait relationships. The correlation coefficient and *P* value were included in each cell. (C–D) Scatter plots displaying the genes in brown (C) and green (D) modules. (E) Volcano plot of the differential expression genes (DEGs) with |log_2_ fold change (FC)| > 1. (F) Venn diagram of intersection genes. (G) The receiver operating characteristic (ROC) curves of the Health/HCC for the testing set in the random forest (RF) and the deep learning (DL) models. (H) Protein-protein interaction (PPI) network analysis of the intersection genes. (I–L) The expression levels of *LCAT* and *CD5L* genes in The Cancer Genome Atlas (TCGA) database (I–J) and Gene Expression Omnibus (GEO) database (K–L). ^***^*P* < 0.001; ME: Module eigengene; WGCNA: weighted gene co-expression network analysis; AUROC: area under the ROC curve.

**Fig. S2.** Development and validation of pseudotargeted lipidomics method. (A) Workflow for constructing the pseudotargeted lipidomics method. (B–C) Representative total ion chromatograms in positive ion mode (B) and negative ion mode (C). (D–E) Corresponding extracted ion chromatograms in positive ion mode (D) and negative ion mode (E). (F–I) Assessment of linearity (F), intra-day repeatability (G), inter-day repeatability (H), and stability (I) in negative ion mode. HPLC-QTOF/MS: high-performance liquid chromatography-tandem quadrupole time-of-flight mass spectrometry; *t*_R_: retention time; CE: collision energy; HPLC-TQMS: HPLC-triple quadrupole MS; Cps: counts per second; RSD: relative standard deviation.

**Fig. S3.** Screen for progressive diagnostic biomarkers to indicate the progression of hepatocellular carcinoma (HCC). (A) Orthogonal partial least squares discriminant analysis (OPLS-DA) score plot for lipid metabolite of “health-hepatitis-cirrhosis-HCC” in positive ion mode (R^2^X=0.674, R^2^Y=0.606, Q^2^=0.329). (B) OPLS-DA score plot for lipid metabolite of “health-hepatitis-cirrhosis-HCC” in negative ion mode (R^2^X=0.633，R^2^Y=0.655，Q^2^=0.523). (C) The receiver operating characteristic (ROC) curves of the Health/Hepatitis/Cirrhosis/HCC for the testing set in the random forest (RF) and the deep learning (DL) models. (D) The value of accuracy, precision, recall, and F1 score in tenfold cross-validation. (E) The Shapley additive explanation (SHAP) values plot of 12 lipids biomarkers. (F) The mean absolute SHAP values plot of 12 lipids biomarkers. (G) Force plots illustrating the contribution of features to the output results of the DL model in predicting the stages of health, hepatitis, cirrhosis, and HCC. The base value was assigned to each group when constructing the DL model. The f(X) value is the model output value used to predict whether the sample is health, hepatitis, cirrhosis, or HCC by comparing it with the base value. The bottom of the plot lists the features, and the area of the color block reflects the SHAP values of the corresponding features. The larger the area, the greater the influence of the feature on the predicted value. The red feature indicates increased risk (to the right), and the blue feature represents reduced risk (to the left). AUROC: area under the ROC curve.

**Fig. S4.** The expression levels of 12 lipid biomarkers across the health, hepatitis, cirrhosis, and hepatocellular carcinoma (HCC) groups. ns: no significance; ^*^ *P* < 0.05; ^**^*P* < 0.01; and ^***^*P* < 0.001.

**Fig. S5.** Receiver operating characteristic (ROC) curves of prediction results based on 12 biomarkers using random forest (RF) and partial least squares discriminant analysis (PLS-DA) models. AUROC: area under the ROC curve; HCC: hepatocellular carcinoma.

**Fig. S6.** Receiver operating characteristic (ROC) curves for the various models. (A–C) ROC curves of Health-Disease (A), Hepatitis-Cirrhosis/HCC (B), and Cirrhosis-HCC (C) for the cohort 1 using established diagnosis models. (D) ROC curve of Health-HCC for the cohort 2 using established diagnosis models. HCC: hepatocellular carcinoma; AUROC: area under the ROC curve.

**Supporting Tables:**

**Table S1** Characteristics of the subjects.

| No. of subjects | Health  (50) | Hepatitis  (30) | Cirrhosis  (30) | HCC  (50) |
| --- | --- | --- | --- | --- |
| Sex | | | | |
| Male | 24 | 14 | 19 | 28 |
| Female | 26 | 16 | 11 | 22 |
| Age | | | | |
| 20–29 | 9 | 2 | 0 | 0 |
| 30–39 | 9 | 3 | 1 | 0 |
| 40–49 | 10 | 7 | 5 | 10 |
| 50–59 | 7 | 14 | 15 | 14 |
| 60–69 | 10 | 3 | 7 | 20 |
| 70–79 | 5 | 0 | 2 | 6 |
| 80–89 | 0 | 1 | 0 | 0 |
| Smoking and drinking status | | | | |
| None | 0 | 0 | 0 | 2 |
| Smoking | 0 | 0 | 0 | 10 |
| Drinking | 0 | 0 | 0 | 10 |
| Both | 0 | 0 | 0 | 8 |
| Unknown | 50 | 30 | 30 | 20 |

HCC: hepatocellular carcinoma.

**Table S2** Correlation coefficient of age, sex, and the patient disease type.

|  | Age | Sex | Patient type |
| --- | --- | --- | --- |
| Age | 1 | –0.003 | 0.128 |
| Sex | –0.003 | 1 | –0.072 |
| Patient type | 0.128 | –0.072 | 1 |

**Table S3** Two-sample T test of sex and age of patients.

|  | t-statistic | DF | Probability > \|t\| |
| --- | --- | --- | --- |
| Assumed homogeneity of variance | 0.035 | 158 | 0 |
| Assumed inhomogeneity of variance | 0.034 | 145 | 0 |

DF: Degree of freedom.

**Table S4** Two-sample T test of age and the disease type of patients.

|  | t-statistic | DF | Probability > \|t\| |
| --- | --- | --- | --- |
| Assumed homogeneity of variance | 0.201 | 158 | 0 |
| Assumed inhomogeneity of variance | 0.183 | 77 | 0 |

DF: Degree of freedom.

**Table S5** Two-sample T test of sex and the disease type of patients.

|  | t-statistic | DF | Probability > \|t\| |
| --- | --- | --- | --- |
| Assumed homogeneity of variance | 0.903 | 158 | 0 |
| Assumed inhomogeneity of variance | 0.902 | 154 | 0 |

DF: Degree of freedom.

**Table S6** Hyperparameters of random forest (RF) models.

| Hyperparameter | Transcriptome | Lipidomics |
| --- | --- | --- |
| n_estimators | 100 | 200 |
| max_depth | None | None |
| criterion | Gini | Gini |
| min_samples_split | 2 | 2 |
| min_samples_leaf | 2 | 1 |

**Table S7** Optimization hyperparameters of deep learning (DL) models.

| Dataset | Model hyperparameters of hidden layers | | |
| --- | --- | --- | --- |
|  | Number of hidden layers | Number of neurons per layer | Activation function per layer |
| Transcriptome | 1 | 21 | Sigmoid |
| Lipidomics | 2 | 20, 20 | Relu, Relu |

**Table S8** HPLC-QTOF/MS parameters.

| Parameter | Positive | Negative |
| --- | --- | --- |
| Gas1 (psi) | 55 | 55 |
| Gas2 (psi) | 55 | 55 |
| Curtain gas (psi) | 30 | 30 |
| Ion source temperature (℃) | 550 | 550 |
| Spray voltage (V) | 5500 | –4500 |
| TOF MS mode scan range (Da) | 50–1500 | 50–1500 |
| Declustering potential (V) | 80 | –80 |
| MS collision energy (V) | 10 | –10 |
| TOF MS/MS scan range (Da) | 50–1500 | 50–1500 |
| MS/MS collision energy (V) | 15、30、45 | –15、–30、–45 |

**Table S9** HPLC-TQMS parameters.

| Parameter | Positive | Negative |
| --- | --- | --- |
| Gas1 (psi) | 55 | 55 |
| Gas2 (psi) | 55 | 55 |
| Curtain gas (psi) | 30 | 30 |
| Ion source temperature (℃) | 550 | 550 |
| Spray voltage (V) | 5500 | –4500 |

**Table S10** Confusion matrix of prediction results based on 12 biomarkers using random forest (RF).

| Sample | Health | Hepatitis | Cirrhosis | HCC | Accuracy |
| --- | --- | --- | --- | --- | --- |
| Health | 29 | 0 | 1 | 0 | 0.967 |
| Hepatitis | 0 | 24 | 6 | 0 | 0.800 |
| Cirrhosis | 0 | 6 | 24 | 0 | 0.800 |
| HCC | 1 | 0 | 0 | 29 | 0.967 |
| Total | 30 | 30 | 31 | 29 | 0.883 |

HCC: hepatocellular carcinoma.

**Table S11** Confusion matrix of prediction results based on 12 biomarkers using partial least squares discriminant analysis (PLS-DA).

| Sample | Health | Hepatitis | Cirrhosis | HCC | Accuracy |
| --- | --- | --- | --- | --- | --- |
| Health | 30 | 0 | 0 | 0 | 1.000 |
| Hepatitis | 0 | 28 | 2 | 0 | 0.933 |
| Cirrhosis | 0 | 0 | 30 | 0 | 1.000 |
| HCC | 0 | 0 | 0 | 30 | 1.000 |
| Total | 30 | 28 | 32 | 30 | 0.983 |

HCC: hepatocellular carcinoma.

**Table S12** Diagnosis models of “health-hepatitis-cirrhosis-HCC” based on 12 lipid biomarkers.

| Diagnosis group | Disease diagnosis models |
| --- | --- |
| Health (+)-Disease (-) | F1= 5.178+0.459*_X1_–*0.678*_X2_–*14.34*_X3_–*16.17*_X4_*+15.147*_X5_–*3.416*_X6_*+0.859*_X7_–*0.64*_X8_–*7.742*_X9_*+3.973*_X10_*+99.587*_X11_*+1.927*_X12_* |
| Hepatitis (+)-Cirrhosis/HCC (-) | F2= 3.287*–*0.17*_X1_*+1.184*_X2_–*0.959*_X3_* +0.539*_X4_–*0.542*_X5_–*3.917*_X6_–*1.209*_X7_*+0.194*_X8_* +0.09*_X9_* +0.657*_X10_–*0.486*_X11_*+0.273*_X12_* |
| Cirrhosis (+)-HCC (-) | F3= –9.284+0.972*_X1_*–0.976*_X2_*–3.66*_X3_–*3.385*_X4_*+3.856*_X5_–*2.135*_X6_*+8.328*_X7_–*0.144*_X8_–*3.862*_X9_*+0.461*_X10_–*11.588*_X11_*+2.841*_X12_* |

+: positive value; -: negative value. X1: FA 20:4;O; X2: TG 50:2;1O|TG 16:0_16:0_18:2;1O; X3: TG 53:8|TG 14:1_21:2_18:5; X4: Cer 45:6;2O|Cer 13:2;2O/32:4; X5: PC 38:4|PC 18:1_20:3; X6: Cer 45:6;4O; X7: TG 54:5;2O|TG 18:1_18:1_18:3;2O; X8: Cer 60:3;4O; X9: Cer 40:2;2O|Cer 18:2;2O/22:0; X10: PC 38:4; X11: FA 18:1;O; X12: Cer 43:0;2O|Cer 18:0;2O/25:0; HCC: hepatocellular carcinoma.

**Table S13** Performance metrics of the diagnostic model in different cohorts.

| Cohort | Diagnosis group | Accuracy | AUROC | AUPRC | Specificity | Sensitivity | F1 Score |
| --- | --- | --- | --- | --- | --- | --- | --- |
| Cohort1 | Health-Disease | 1.000 | 1.000 | 1.000 | 1.000 | 1.000 | 1.000 |
|  | Hepatitis-Cirrhosis/HCC | 0.889 | 0.867 | 0.981 | 0.903 | 0.933 | 0.918 |
|  | Cirrhosis-HCC | 1.000 | 1.000 | 1.000 | 1.000 | 1.000 | 1.000 |
| Cohort2 | Health-HCC | 0.925 | 0.925 | 0.949 | 0.947 | 0.900 | 0.923 |

AUROC: area under the receiver operating characteristic (ROC) curve; AUPRC: area under the precision-recall curve (PRC); HCC: hepatocellular carcinoma.

**Table S14** The confusion matrix of prediction results in cohort 2.

| Sample | Health | HCC | Accuracy |
| --- | --- | --- | --- |
| Health | 19 | 0 | 0.975 |
| Hepatitis | 1 | 0 | – |
| Cirrhosis | 0 | 2 | – |
| HCC | 0 | 18 | 0.900 |
| Total | 20 | 20 | 0.925 |

–: No data; HCC: hepatocellular carcinoma.
